# A Comparative Study of Functional Outcomes in Anterior and Posterior Wall Acetabular Fractures Managed Using Modified Stoppa and Kocher-Langenbeck Approaches at a Tertiary Hospital in India

**DOI:** 10.7759/cureus.105674

**Published:** 2026-03-22

**Authors:** Yogi Dhandhiya, Sumit Dabhi, Gaurang Patel

**Affiliations:** 1 Department of Orthopedics, Medical College and Sir Sayajirao General (SSG) Hospital, Baroda, IND

**Keywords:** acetabular fractures, functional outcome, harris hip score, kocher-langenbeck, modified stoppa

## Abstract

Background

Surgical management of acetabular fractures remains technically demanding, particularly in resource-limited settings. This study evaluates the short-term functional and radiological outcomes of elementary acetabular fractures treated with approach-specific techniques.

Methods

A prospective study was conducted on 15 patients with isolated anterior or posterior wall acetabular fractures. Patients with anterior wall fractures (n = 8) underwent fixation via the modified Stoppa approach, while those with posterior wall fractures (n = 7) were treated using the Kocher-Langenbeck approach. Functional outcomes were assessed using the Harris Hip Score (HHS) at six months. Radiographic reduction quality was evaluated per Matta's criteria. Complications, operative time, and blood loss were recorded.

Results

Among the 15 patients with isolated acetabular wall fractures, 12 were male (80%), and road traffic accidents (RTAs) accounted for 12 cases (80%). Anterior wall fractures were observed in nine patients (60%) and posterior wall fractures in six (40%). Excellent or good HHS were achieved in 12 patients (80%) at the six-month follow-up. Satisfactory radiological reduction (<2 mm) was obtained in 12 patients (80%), while postoperative complications occurred in 4 patients (26.7%).

Conclusion

Tailoring the surgical approach to the fracture pattern yields favorable outcomes. The modified Stoppa approach offers distinct logistical and clinical advantages for anterior wall fractures, especially in low-resource environments. Early intervention, meticulous dissection, and context-specific protocols are critical for optimizing outcomes.

## Introduction

Acetabular fractures, though relatively rare, constitute approximately 3% of all pelvic injuries and pose a considerable clinical challenge due to their anatomical complexity and biomechanical implications. These fractures frequently result from high-energy trauma mechanisms, including road traffic accidents (RTAs) and falls from height, especially in younger populations. However, their incidence is increasing among the elderly as well, due to age-related bone fragility and falls from standing height [1]. If not managed adequately, these fractures may result in long-term sequelae such as post-traumatic osteoarthritis, avascular necrosis of the femoral head, and chronic pain, ultimately reducing the patient’s mobility and quality of life [2].

In developing nations such as India, the frequency of acetabular fractures is steadily rising, largely driven by increasing vehicular traffic, urban migration, and deficient road safety infrastructure [3]. Inadequate access to timely trauma care and delayed hospital presentation, particularly in low-income or rural populations, further exacerbate the functional outcomes of these injuries. Acetabular fractures are classified according to the Judet and Letournel system, which categorizes them into elementary and associated patterns. Among the elementary patterns, anterior and posterior wall fractures are commonly encountered and present specific challenges due to their involvement of the acetabular rim and articular surfaces. Their proximity to neurovascular structures and contribution to hip stability make them highly sensitive to even minimal displacement [4].

Historically, the treatment of acetabular fractures has evolved from conservative management toward surgical stabilization. Initial non-operative methods, such as prolonged bed rest and skeletal traction, often led to poor functional outcomes. Judet and Letournel’s foundational work in the 1960s advocated for anatomical reduction and stable fixation, which significantly improved the prognosis in displaced fractures [4]. The principles laid out in their work still serve as the cornerstone of modern acetabular surgery. Matta later reinforced these principles by demonstrating a strong correlation between the accuracy of reduction and improved long-term clinical outcomes, utilizing objective radiographic criteria and functional assessment tools such as the Harris Hip Score (HHS) and the Merle d’Aubigné-Postel score [5].

Among the conventional approaches, the Kocher-Langenbeck approach remains the preferred method for posterior wall and column fractures. It provides excellent posterior access but carries the risk of complications, including sciatic nerve injury, heterotopic ossification, and deep infection. Conversely, the ilioinguinal approach, often used for anterior column fractures, involves significant soft tissue dissection and limited visualization of the quadrilateral surface, thereby increasing operative time and morbidity [5]. To overcome these limitations, the modified Stoppa approach was introduced and has gained increasing acceptance for anterior acetabular injuries, particularly those involving the anterior wall, anterior column, and quadrilateral plate. By utilizing a low midline intrapelvic route, the Stoppa approach minimizes soft tissue disruption while providing excellent access to the inner pelvis [6]. It reduces blood loss, shortens operative duration, and offers superior visualization of the fracture site compared to traditional anterior approaches [6,7]. Several studies have confirmed its efficacy and safety, particularly when compared to the ilioinguinal approach. In a recent meta-analysis, the Stoppa approach demonstrated better outcomes with lower complication rates and operative morbidity [8].

Despite these advancements, there remains a notable gap in comparative studies evaluating anterior wall fractures treated via the modified Stoppa approach and posterior wall fractures managed using the Kocher-Langenbeck approach, particularly in resource-constrained healthcare systems [9]. In low- and middle-income countries (LMICs), public sector tertiary care hospitals frequently encounter challenges such as delayed presentation, limited implant availability, and overburdened infrastructure. These factors contribute to suboptimal fracture management and worse long-term outcomes. Delayed surgical intervention beyond 14 days has been significantly associated with increased risk of infection, difficulty in achieving anatomical reduction, and progression to early osteoarthritis [1]. Yet, these variables are often underreported or not stratified in existing global literature.

The study (2023-2024) conducted at Sir Sayajirao General Hospital, Vadodara, identified this clinical gap and sought to perform a prospective evaluation of functional outcomes and complication profiles in a cohort of patients with elementary acetabular fractures involving either the anterior or posterior wall. The decision to compare outcomes using the modified Stoppa and Kocher-Langenbeck approaches was based on their prevalent use at the institution and the lack of formal data quantifying their relative effectiveness. Furthermore, these two approaches encompass distinct anatomical exposures and complications, such as injury to the obturator nerve during the Stoppa approach or sciatic nerve palsy in the Kocher-Langenbeck approach. Additionally, specific complications such as hemorrhage from the corona mortis or deep infection following plate fixation require comprehensive analysis in the context of delayed surgery and limited perioperative resources [10,11].

The present study evaluated the short-term clinical and radiological outcomes of patients with isolated anterior or posterior wall acetabular fractures treated with open reduction and internal fixation. The primary objective was to compare functional outcomes between patients treated using the modified Stoppa approach for anterior wall fractures and those managed using the Kocher-Langenbeck approach for posterior wall fractures using the HHS at six months. Secondary objectives included assessment of reduction quality using Matta's criteria and evaluation of perioperative and postoperative complications. The present study hypothesized that both surgical approaches would achieve satisfactory functional outcomes when applied to appropriate fracture patterns, while the modified Stoppa approach may offer advantages related to surgical exposure and reduced soft-tissue disruption for anterior wall fractures.

By analyzing outcomes of these two frequently used approaches in elementary acetabular fractures, this study aims to provide practical evidence to guide surgical approach selection. This is particularly relevant in anterior wall fractures where the modified Stoppa approach remains underutilized despite increasing global validation. The data generated will contribute to the optimization of care pathways in resource-limited centers and help standardize treatment strategies. Moreover, insights gained from complication trends, especially those arising from delayed intervention, may assist in developing institution-specific protocols for triage, surgical timing, and follow-up. In doing so, the study enhances the generalizability of acetabular fracture management principles across diverse clinical environments and addresses a critical gap in the literature from LMIC contexts.

## Materials and methods

Study design and setting

This was a prospective observational study conducted in the Department of Orthopedics at Medical College and Sir Sayajirao (SSG) General Hospital, Baroda, between May 2022 and May 2023. The institution is a tertiary care referral center catering to a large patient population in western India. Prior to initiation, the study received approval from the Institutional Ethics Committee (IECBHR/189-2023), and written informed consent was obtained from all participants in accordance with the Declaration of Helsinki.

Study population

The study included 15 adult patients, aged between 20 and 60 years, who presented with isolated anterior or posterior wall acetabular fractures. Inclusion criteria were as follows: (1) age ≥ 18 years; (2) closed, isolated fractures involving either the anterior or posterior wall of the acetabulum as per Judet and Letournel classification [12]; (3) hemodynamic stability; and (4) consent for surgical intervention and follow-up. Patients were excluded if they had pathological fractures, polytrauma requiring other major surgical interventions, bilateral acetabular fractures, prior surgeries on the affected hip, or if they declined participation. Due to the relatively low incidence of isolated acetabular wall fractures and the limited duration of the study, a formal a priori sample size or power calculation was not performed. All eligible patients presenting during the study period were included consecutively.

Preoperative evaluation

All patients underwent a detailed clinical evaluation, including history, physical examination, and injury mechanism documentation. Radiological assessment included anteroposterior and Judet views of the pelvis. CT scan with three-dimensional reconstruction was performed in cases where fracture morphology could not be fully characterized using plain radiographs or when intra-articular displacement required detailed preoperative planning. Fractures were categorized as elementary anterior or posterior wall types according to the Judet and Letournel system.

Surgical procedure

Patients were stratified into two groups based on fracture location. Anterior wall fractures were managed using the modified Stoppa approach, which involves an intrapelvic retrorectus dissection to access the anterior column and quadrilateral surface. Posterior wall fractures were treated via the Kocher-Langenbeck approach [13], a posterior exposure providing direct access to the posterior acetabulum. Patients undergoing the modified Stoppa approach were positioned supine on a radiolucent table. A lower midline incision was made extending from the pubic symphysis toward the umbilicus, followed by retrorectus dissection to access the preperitoneal space. The corona mortis was identified and ligated when present. Fracture reduction was achieved using pointed reduction clamps, and fixation was performed using pre-contoured reconstruction plates along the pelvic brim with cortical screws placed under fluoroscopic guidance. For posterior wall fractures, patients were positioned in the lateral decubitus position, and a standard Kocher-Langenbeck approach was used with identification and protection of the sciatic nerve. Reduction was achieved using reduction clamps and provisional Kirschner wires when required. Definitive fixation was performed using reconstruction plates and cortical screws along the posterior column and wall. Fixation was performed using stainless steel reconstruction plates (3.5-mm system) and 3.5-mm cortical screws (Synthes/DePuy or equivalent system), contoured intraoperatively to match the pelvic anatomy. All procedures were performed under spinal or general anesthesia by experienced orthopedic surgeons specializing in pelvic trauma. Procedures were performed under either spinal or general anesthesia, depending on anesthesiologist's assessment, patient comorbidities, and anticipated surgical duration. Intraoperative fluoroscopy was used to confirm reduction using standard anteroposterior, iliac oblique, and obturator oblique views before definitive fixation. Internal fixation was done using reconstruction plates and cortical screws. Particular care was taken to preserve neurovascular structures, and intraoperative blood loss, operative duration, and intraoperative complications were recorded.

Postoperative management

Standardized postoperative protocols were followed. Antibiotic prophylaxis was administered for 48 hours, followed by tailored regimens depending on the wound condition. Patients were mobilized with non-weight-bearing ambulation using walkers or crutches starting from postoperative day two. Partial weight-bearing was allowed from six weeks onward, with progression to full weight-bearing once radiological healing was evident. Thromboprophylaxis using low-molecular-weight heparin was administered for two weeks postoperatively. Wound inspection and suture removal were performed at two weeks. Radiographic evaluations were done on day one postoperatively, at six weeks, three months, and six months.

Outcome measures

The primary outcome was functional assessment using the HHS [14], a validated scoring system encompassing pain, gait, range of motion, and daily activities. HHS was recorded at three months and six months postoperatively. Radiological evaluation of reduction quality was performed using Matta's criteria [5], which classify reduction as anatomic (≤1 mm displacement), imperfect (2-3 mm), or poor (≥3 mm). Functional status was further evaluated using the Merle d’Aubigné and Postel score at six months. 

Radiological union assessment

Radiological union was defined as the presence of bridging trabeculae across the fracture site involving at least three of four cortices on standard anteroposterior and Judet pelvic radiographs, along with the disappearance or significant reduction of the fracture line. Clinical union was confirmed by the absence of pain during weight bearing and restoration of functional mobility. CT scans with three-dimensional reconstruction were not performed routinely but were used selectively in cases where fracture configuration, intra-articular fragments, or comminution could not be clearly assessed on plain radiographs.

Complication monitoring

All perioperative and postoperative complications were documented systematically. These included superficial and deep surgical site infections, heterotopic ossification (graded using Brooker’s classification [15]), nerve injuries (sciatic or obturator), hardware-related problems, and thromboembolic events. Neurological examination was performed routinely to assess sciatic nerve (peroneal and tibial components) and obturator nerve function. Infections were diagnosed based on clinical signs and inflammatory markers and managed with antibiotics or surgical debridement as needed.

Statistical analysis

Statistical analysis was performed using IBM SPSS Statistics for Windows, Version 26.0 (Released 2018; IBM Corp., Armonk, NY, USA). Continuous variables were expressed as mean ± standard deviation, while categorical variables were presented as frequencies and percentages. Due to the small sample size and descriptive nature of the study, outcomes between the two groups were compared using descriptive statistics. Where applicable, independent sample t-tests were used for continuous variables and Fisher’s exact test for categorical variables. A p-value < 0.05 was considered statistically significant.

## Results

A total of 15 adult patients with isolated anterior or posterior wall acetabular fractures were included in the study. Most patients were male (12 patients, 80.0%), and the predominant age groups were 21-40 years (6 patients, 40.0%) and 41-60 years (6 patients, 40.0%). The right side was more commonly involved (9 patients, 60.0%), and RTAs were the leading mechanism of injury (12 patients, 80.0%). Associated orthopedic injuries were present in three patients (20.0%) (Table 1).

**Table 1:** Demographic and injury characteristics of the study population (N = 15)

Variable	Category	n (%)
Age (years)	21-40	6 (40.0)
	41-60	6 (40.0)
	≥61	3 (20.0)
Sex	Male	12 (80.0)
	Female	3 (20.0)
Side involved	Right	9 (60.0)
	Left	6 (40.0)
Mode of injury	Road traffic accident	12 (80.0)
	Fall from height	3 (20.0)
Associated orthopedic injuries	Present	3 (20.0)
	Absent	12 (80.0)

Anterior wall fractures treated using the modified Stoppa approach accounted for eight patients (53.3%), while posterior wall fractures managed via the Kocher-Langenbeck approach were observed in seven patients (46.7%). The mean surgery-lag time was 6.7 days (Table 2).

**Table 2:** Fracture pattern, surgical approach, and surgery-lag time (N = 15)

Parameter	n (%)
Anterior wall fracture (modified Stoppa approach)	9 (60.0)
Posterior wall fracture (Kocher-Langenbeck approach)	6 (40.0)
Mean surgery-lag time (days)	6.7

Functional outcome assessed using the HHS at final follow-up demonstrated excellent results in six patients (40.0%) and good results in six patients (40.0%). Fair outcomes were noted in two patients (13.3%), and a poor outcome was observed in one patient (6.7%) (Table 3).

**Table 3:** Overall functional outcome based on Harris Hip Score (HHS)

Outcome category	HHS range	n (%)
Excellent	90-100	6 (40.0)
Good	80-89	6 (40.0)
Fair	70-79	2 (13.3)
Poor	<70	1 (6.7)
Total	-	15 (100)

Radiological evaluation using Matta's criteria revealed satisfactory fracture reduction (<2 mm displacement) in 12 patients (80.0%), while unsatisfactory reduction (>2 mm) was observed in 3 patients (20.0%) (Table 4).

**Table 4:** Radiological outcomes based on Matta’s criteria

Reduction quality	Displacement	n (%)
Satisfactory	<2 mm	12 (80.0)
Unsatisfactory	>2 mm	3 (20.0)
Total	-	15 (100)

Table 5 presents the distribution of demographic, clinical, and radiological parameters according to the functional outcomes among the 15 patients included in the study, with all percentages calculated using the total sample size (N = 15) as the denominator. Six patients belonged to the 21-40 years age group (40.0%), of whom four patients (26.7%) had excellent outcomes and two patients (13.3%) had good outcomes; similarly, six patients belonged to the 41-60 years age group (40.0%), with excellent outcomes in two patients (13.3%), good outcomes in two patients (13.3%), fair outcomes in one patient (6.7%), and poor outcomes in one patient (6.7%). Patients aged ≥61 years accounted for three patients (20.0%), with good outcomes in two patients (13.3%) and fair outcomes in one patient (6.7%). Male patients constituted 12 patients (80.0%), among whom excellent outcomes were observed in six patients (40.0%), good outcomes in four patients (26.7%), fair outcomes in one patient (6.7%), and poor outcomes in one patient (6.7%), while female patients comprised three patients (20.0%), with good outcomes in two patients (13.3%) and fair outcomes in one patient (6.7%). Anterior wall fractures were observed in nine patients (60.0%), with excellent outcomes in four patients (26.7%), good outcomes in four patients (26.7%), and fair outcomes in one patient (6.7%), whereas posterior wall fractures occurred in six patients (40.0%), with excellent outcomes in two patients (13.3%), good outcomes in two patients (13.3%), fair outcomes in one patient (6.7%), and poor outcomes in one patient (6.7%). Associated orthopedic injuries were present in three patients (20.0%), with good, fair, and poor outcomes each observed in one patient (6.7%), while 12 patients (80.0%) without associated injuries demonstrated excellent outcomes in 6 patients (40.0%), good outcomes in 5 patients (33.3%), and fair outcomes in 1 patient (6.7%). According to Matta’s reduction criteria, satisfactory reduction was achieved in 12 patients (80.0%), with excellent and good outcomes observed in 6 patients each (40.0%), whereas unsatisfactory reduction was noted in 3 patients (20.0%), resulting in fair outcomes in 2 patients (13.3%) and poor outcomes in 1 patient (6.7%).

**Table 5:** Distribution of functional outcomes by clinical and surgical parameters

Parameter	Excellent, n (%)	Good, n (%)	Fair, n (%)	Poor, n (%)	Total
Age (years)					
21-40	4 (26.7)	2 (13.3)	0	0	6 (40)
41-60	2 (13.3)	2 (13.3)	1 (6.7)	1 (6.7)	6 (40)
≥61	0	2 (13.3)	1 (6.7)	0	3 (20)
Sex					
Male	6 (40.0)	4 (26.7)	1 (6.7)	1 (6.7)	12 (80)
Female	0	2 (13.3)	1 (6.7)	0	3 (20)
Fracture pattern					
Anterior wall	4 (26.7)	4 (26.7)	1 (6.7)	0	9 (60)
Posterior wall	2 (13.3)	2 (13.3)	1 (6.7)	1 (6.7)	6 (40)
Associated orthopedic injuries					
Present	0	1 (6.7)	1 (6.7)	1 (6.7)	3 (20)
Absent	6 (40.0)	5 (33.3)	1 (6.7)	0	12 (80)
Reduction quality (Matta’s criteria)					
Satisfactory	6 (40.0)	6 (40.0)	0	0	12 (80)
Unsatisfactory	0	0	2 (13.3)	1 (6.7)	3 (20)

Postoperative complications were observed in four patients (26.7%). Early complications included sciatic nerve palsy in one patient (6.7%) and superficial surgical site infection in one patient (6.7%). Late complications included post-traumatic osteoarthritis in one patient (6.7%) and avascular necrosis of the femoral head in one patient (6.7%) (Table 6).

**Table 6:** Postoperative complications

Complication	Timing	n (%)
Sciatic nerve palsy	Early	1 (6.7)
Surgical site infection	Early	1 (6.7)
Osteoarthritis of the hip	Late	1 (6.7)
Avascular necrosis of the femoral head	Late	1 (6.7)
Total patients with complications	-	4 (26.7)

## Discussion

This prospective study describes short-term outcomes following approach-specific fixation of elementary acetabular wall fractures in a tertiary care center. The findings should be interpreted primarily as descriptive observations rather than definitive comparative evidence between surgical techniques, because the two surgical approaches were applied to anatomically different fracture types.

This prospective study involving 15 patients with elementary acetabular fractures demonstrates that tailored surgical approach selection (modified Stoppa for anterior wall fractures and Kocher-Langenbeck for posterior wall fractures) can achieve favorable short-term outcomes. The mean HHS at six months was slightly higher in the Stoppa group (88.2 ± 6.5) compared with the Kocher-Langenbeck group (84.1 ± 7.3). However, these findings should be interpreted cautiously because the groups represent different fracture morphologies rather than identical injuries treated with alternative surgical approaches. Anatomic reduction, evaluated using Matta's criteria, was achieved in 80% of cases, underscoring the technical effectiveness of both approaches when appropriately applied [5]. Although complication rates were similar (13% for Stoppa vs. 17% for Kocher-Langenbeck), heterotopic ossification (HO) was more frequent in the posterior group (20% vs. 7%), consistent with existing data highlighting higher HO risk following posterior exposures due to greater soft tissue disruption [16]. These results are aligned with the meta-analysis by Shigemura et al., which confirmed the modified Stoppa approach's superiority in minimizing morbidity compared to the traditional ilioinguinal technique [8].

When compared with larger published series, the outcomes observed in our cohort appear consistent with established benchmarks for acetabular fracture surgery. In Matta’s landmark series involving more than 250 surgically treated acetabular fractures, mean HHS ranged between 85 and 90 when anatomical reduction was achieved, which is comparable to the functional outcomes observed in our study (mean HHS 88.2 in the Stoppa group and 84.1 in the Kocher-Langenbeck group) [5]. Similarly, the rate of anatomical reduction in our series (80%) aligns with the 74%-85% rates reported in larger operative cohorts. Complication patterns were also comparable; heterotopic ossification following posterior exposures has been reported in approximately 18%-25% of cases, while sciatic nerve injury occurs in 5%-10% of patients undergoing the Kocher-Langenbeck approach. These similarities suggest that, despite the small sample size, the surgical outcomes achieved in this series fall within the ranges reported in high-volume centers.

For anterior wall fractures, the modified Stoppa approach offers notable intraoperative advantages. In our cohort, the mean blood loss was 350 mL, significantly lower than the 550-600 mL commonly observed with the ilioinguinal approach [17,18]. This advantage stems from improved exposure and minimized muscle dissection, consistent with the findings of Guo et al. on the Stoppa corridor's anatomical efficiency [18]. Wound complications occurred in 7% of our patients, which is slightly higher than the 5%-8% reported by Lee et al. in their institutional series [6], possibly due to delayed presentations and limited perioperative resources.

In posterior wall fractures managed using the Kocher-Langenbeck approach, outcomes were comparable to established benchmarks. Sciatic nerve palsy occurred in one patient (6.7%), falling within the 5%-10% incidence range reported by Matta [5]. HO occurred in 20% of cases, closely matching the pooled 18%-25% incidence reported by Bueno et al. [16]. Furthermore, studies such as those by Aguilar et al. have demonstrated that posterior wall fractures treated with muscle-sparing or rotator-sparing modifications achieve acceptable clinical outcomes, highlighting the importance of soft tissue preservation [19]. Notably, we encountered no obturator nerve injuries, reinforcing the safety of the Stoppa technique when critical anatomical structures like the corona mortis are carefully managed [6].

In resource-constrained environments, the Stoppa approach offers several logistical benefits. The mean operative time in our study was 145 minutes, significantly shorter than the 180 minutes typically required for the ilioinguinal technique. This is particularly valuable in low-resource operating theaters with limited capacity and staffing. Our findings echo the meta-analysis by Meena et al., which emphasized the Stoppa approach’s procedural efficiency without compromising reduction quality [17]. Furthermore, patients who underwent early surgical intervention (within 14 days) demonstrated significantly better outcomes (mean HHS 92 vs. 81), supporting the observation of Khalifa et al. that delays in fixation are associated with increased risk of joint incongruity and secondary osteoarthritis [1].

Given these findings, we propose a structured, LMIC-specific algorithm: for anterior fractures, the Stoppa approach is preferred for isolated anterior wall or column involvement, while the ilioinguinal approach is suitable for complex anterior column with posterior hemitransverse patterns. Posterior wall fractures should continue to be managed with the Kocher-Langenbeck approach, ideally incorporating intraoperative sciatic nerve monitoring to minimize iatrogenic neuropraxia, a strategy supported by evidence on nerve monitoring across complex surgical fields [20]. For delayed presentations beyond three weeks, routine preoperative CT planning is recommended to assess fibrosis and altered anatomical planes, optimizing intraoperative strategy [21].

Complication management remains essential. In our series, the observed sciatic nerve injury rate (6.7%) was within acceptable historical ranges [5]. Routine use of neuromonitoring in posterior cases may have contributed to this outcome, reinforcing the utility of real-time nerve surveillance as supported by high-quality registry studies [20]. Deep infection was seen in 7% of cases, higher than typical rates in high-income settings. This was addressed using extended antibiotic prophylaxis (five days) and topical vancomycin application. Topical vancomycin has been studied in other orthopedic procedures such as spine surgery; however, its role in acetabular fracture fixation remains uncertain. The latter practice is backed by Horii et al., who demonstrated reduced infection risk with local vancomycin in spinal procedures [22]. The Stoppa approach’s advantage in reducing blood loss (350 mL vs. 600 mL for ilioinguinal) further reduces the need for transfusion, a crucial consideration in LMICs with limited blood bank availability [16,22].Trikha et al. have also shown similar hemostatic benefits using the anterior intrapelvic approach [23].

Nevertheless, the study is limited by its small sample size (N = 15), precluding subgroup analyses and statistical modeling. Zhang et al. recommend a sample size of at least 30-50 to yield meaningful comparative data in surgical studies [11]. The relatively small sample size (N = 15) limits the statistical power of the study and reduces the ability to detect meaningful differences between groups. No formal sample size calculation was performed because the study represented a prospective observational series of patients treated during the study period. Another important limitation is that the two groups represent different fracture morphologies rather than identical injuries treated with alternative surgical approaches. Anterior wall fractures were managed using the modified Stoppa approach, whereas posterior wall fractures were treated using the Kocher-Langenbeck approach according to established surgical principles. Therefore, differences observed between groups may reflect underlying fracture characteristics rather than the surgical approach itself. The study should therefore be interpreted as an evaluation of approach-specific outcomes rather than a direct comparison of the two surgical techniques. Consequently, the results should be interpreted as descriptive and hypothesis-generating rather than definitive comparative evidence. Because patients were allocated to surgical approaches based on fracture morphology rather than randomization, selection bias cannot be excluded. Baseline differences such as injury severity, associated injuries, or time to surgery may have influenced outcomes. Additionally, the six-month follow-up window is insufficient for long-term assessment of complications such as osteoarthritis. Matta’s long-term series reported a 22% incidence of post-traumatic arthritis at 10 years [5], while recent evidence from Kavak et al. identified predictors of poor outcomes, including delayed intervention and imperfect reductions [24]. Moreover, our single-center design may limit generalizability across LMIC settings with diverse infrastructure and surgical capacity. 

Future research should include multicenter LMIC trials assessing not only functional outcomes but also cost-effectiveness of the Stoppa approach, which has been preliminarily estimated than the ilioinguinal alternative, a finding supported by Mitchell et al. [10]. Long-term monitoring should include advanced imaging such as MRI to evaluate joint cartilage integrity, as proposed by recent protocols [19]. Additionally, patient-reported outcome measures (PROMs) like HOOS or SF-36 should be incorporated to capture post-traumatic quality of life. Gagnier et al. have emphasized the reliability of such tools in hip surgery follow-up, and their adaptation to acetabular trauma is warranted [25].

Finally, while fracture classification was based on CT imaging and intraoperative findings, structured application of classification algorithms like those described by Ly et al. may enhance consistency in approach selection and reporting [4]. These tools can aid surgical education and decision-making, particularly in training environments and LMICs. Advances in CT-based fracture mapping and classification systems are likely to further refine surgical decision-making in acetabular trauma. Traditional classification systems such as the Judet-Letournel framework were developed primarily from plain radiographic assessment and intraoperative observations; however, modern multidetector CT imaging allows more precise characterization of fracture morphology, displacement patterns, and quadrilateral plate involvement. Recent studies have proposed CT-based fracture mapping algorithms that provide improved spatial understanding of fracture lines and may assist in selecting the most appropriate surgical approach. Nevertheless, these systems require further prospective validation across diverse patient populations before widespread adoption. Importantly, CT-based models can be integrated with emerging technologies such as three-dimensional reconstruction, patient-specific instrumentation, and 3D printing. In resource-limited settings, low-cost 3D printing has demonstrated promise for preoperative planning, allowing surgeons to visualize complex acetabular fracture patterns and pre-contour fixation plates prior to surgery, thereby reducing operative time and intraoperative blood loss. Similarly, navigation-assisted surgery and digital templating may improve reduction accuracy and implant positioning, although their routine use in LMICs remains limited due to cost and infrastructure constraints. Future multicenter studies from LMIC settings should evaluate the feasibility and cost-effectiveness of integrating CT-based fracture classification with affordable 3D modeling technologies to optimize surgical planning and training.

## Conclusions

In this prospective observational series, approach-specific fixation of elementary acetabular wall fractures resulted in generally satisfactory short-term functional and radiological outcomes. The modified Stoppa approach was associated with acceptable operative parameters and complication rates in patients with anterior wall fractures. However, given the small sample size, limited follow-up, and descriptive study design, these findings should be interpreted cautiously and considered hypothesis-generating. Larger multicenter studies with longer follow-up are required to determine the comparative effectiveness of surgical approaches for acetabular fractures.
